# iCut: an Integrative Cut Algorithm Enables Accurate Segmentation of Touching Cells

**DOI:** 10.1038/srep12089

**Published:** 2015-07-14

**Authors:** Yong He, Hui Gong, Benyi Xiong, Xiaofeng Xu, Anan Li, Tao Jiang, Qingtao Sun, Simin Wang, Qingming Luo, Shangbin Chen

**Affiliations:** 1 Britton Chance Center for Biomedical Photonics, Huazhong University of Science and Technology-Wuhan National Laboratory for Optoelectronics, Wuhan, Hubei, China; 2 MoE Key Laboratory for Biomedical Photonics, Department of Biomedical Engineering, Huazhong University of Science and Technology, Wuhan, Hubei, China

## Abstract

Individual cells play essential roles in the biological processes of the brain. The number of neurons changes during both normal development and disease progression. High-resolution imaging has made it possible to directly count cells. However, the automatic and precise segmentation of touching cells continues to be a major challenge for massive and highly complex datasets. Thus, an integrative cut (iCut) algorithm, which combines information regarding spatial location and intervening and concave contours with the established normalized cut, has been developed. iCut involves two key steps: (1) a weighting matrix is first constructed with the abovementioned information regarding the touching cells and (2) a normalized cut algorithm that uses the weighting matrix is implemented to separate the touching cells into isolated cells. This novel algorithm was evaluated using two types of data: the open SIMCEP benchmark dataset and our micro-optical imaging dataset from a Nissl-stained mouse brain. It has achieved a promising recall/precision of 91.2 ± 2.1%/94.1 ± 1.8% and 86.8 ± 4.1%/87.5 ± 5.7%, respectively, for the two datasets. As quantified using the harmonic mean of recall and precision, the accuracy of iCut is higher than that of some state-of-the-art algorithms. The better performance of this fully automated algorithm can benefit studies of brain cytoarchitecture.

Cytoarchitecture is the basis of brain function^1^. The number of neurons changes during both normal development^2^ and disease progression^3^. A better understanding of brain function and diseases requires the refinement of techniques for cell counting and morphology^4^. Benefiting from advanced light microcopy, researchers have obtained massive image datasets at cellular resolution from large-scale brain samples and even a whole mouse brain^5^. Rapid and accurate segmentation of individual cells is urgently needed for quantitative analysis^6^. Because some cells may touch or overlap^7^, the separation of densely clustered cells remains an open problem. In practice, manually identifying the positions and extracting the contours of millions of cells is error prone and time consuming. It is crucial to develop an automatic algorithm to detect and segment large number of cells, especially touching cells.

Many automated methods for detecting and segmenting isolated cells have already been developed, but these algorithms are not applicable to touching cells. Other approaches have been proposed to address this specific issue^8^. For example, the widely used watershed algorithm can segment touching cells but easily causes over-segmentation. Another algorithm, the coupled level set algorithm, can avoid over-segmentation and can also separate touching cells when an initial contour is manually set for each cell^9^,^10^. However, this manual step is impractical for a biological image containing millions of cells. The iterative radial voting method, which relies on gradient projection, can rapidly detect the centroids of touching cells^11^,^12^,^13^, but it depends heavily on the parameter settings and cell size. The graph-cut and multi-scale LoG filtering algorithm has partially overcome the problems of varying size and cell contiguity^8^, but it is sensitive to heterogeneous brightness. Recently, an attributed relational graph algorithm was proposed^14^; this algorithm uses four types of primitives to construct an attributed relational graph to segment touching cells. However, this algorithm relies on the orientation of the *Sobel* operator and produces an inaccurate orientation in a region with heterogeneous brightness. All the aforementioned algorithms require a grayscale image. Utilizing geometric characteristics to segment touching cells, a series of concave point detection methods^15^,^16^,^17^,^18^,^19^ were developed by directly linking the concave points between touching cells. These methods must construct rigorous linking rules, making it difficult to use these methods to segment multiple touching cells. Thus far, the segmentation of touching cells has been a challenge for many methods and has remained a bottleneck in the high-throughput analysis of brain anatomy^20^.

This paper describes an integrative cut (iCut) algorithm that combines information regarding spatial location and the intervening and concave contours with a normalized cut algorithm for the accurate segmentation of cells. Here, the intervening and concave contours are the boundary and concave points, respectively, on the edge of a target cell. For the first time, the aforementioned information regarding touching cells is encoded when constructing the weighting matrix of the normalized cut algorithm. Tested with both simulated and experimental image datasets, the iCut algorithm enables the automatic and precise segmentation of touching cells. This algorithm can facilitate high-throughput analysis of brain anatomy using massive image datasets.

## Results

### Cell segmentation of SIMCEP benchmark and micro-optical images

We ran the iCut algorithm on three datasets (for details, see the Methods section): the SIMCEP Benchmark_Set1 dataset^21^, a micro-optical dataset obtained using a Nissl-staining and micro-optical sectioning tomography imaging system^22^ (NSMOST) and the conventional Nissl-staining mouse brain slice dataset at brainmaps.org^23^. Only the first two datasets were used for quantitative evaluation of the iCut algorithm. The SIMCEP Benchmark_Set1 dataset (synthetic dataset) contained 20 images, named SIM1 to SIM20, and the NSMOST dataset (real dataset) contained 20 images, named NS1 to NS20. The last dataset was used as a pilot test of iCut with a real tissue sample.

**Figure 1:**
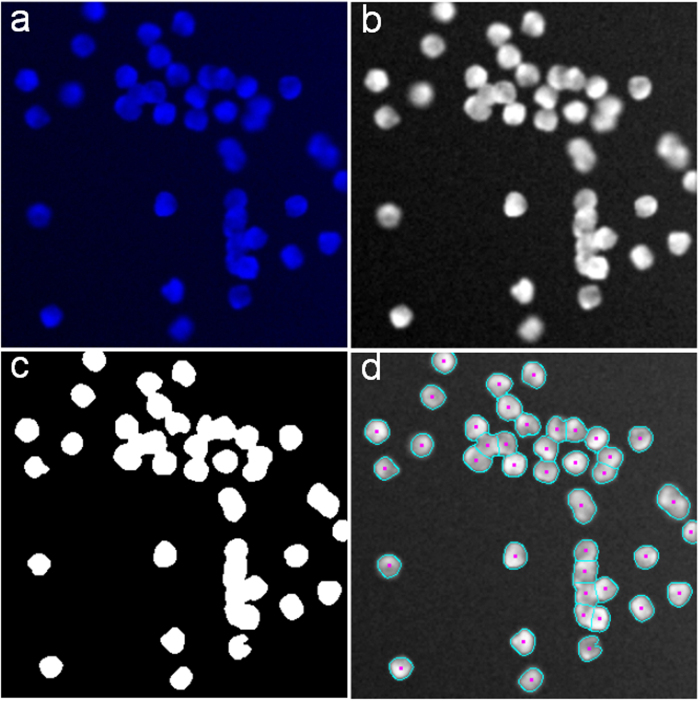
Cell segmentation result of a sub-image of the SIM1 image from the SIMCEP Benchmark_Set1 dataset. (**a**) A sub-image of the SIM1 image, which is 450 × 450 pixels. (**b**) The grayscale image of the sub-image. (**c**) The binary image obtained by image preprocessing. (**d**) The iCut segmentation results. The cyan lines are the boundaries of partitioned groups. The pink points are the centroids of the groups and are also the cell centroids.

**Figure 2:**
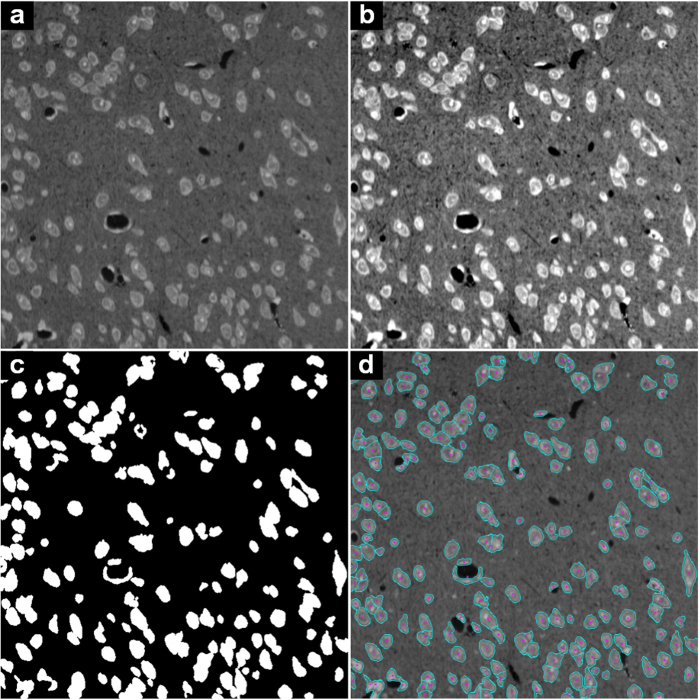
Cell segmentation result of the NS1 image of the NSMOST dataset. (**a**) The original grayscale image. (**b**) The image enhanced by linear stretching. (**c**) The binary image obtained by preprocessing. (**d**) The iCut segmentation results. The cyan lines are the boundaries of the partitioned groups. The pink points are the centroids of the groups and are also the cell centroids.

Typical results are shown in Figs 1 and 2. A sub-image of the SIM1 image is shown in Fig. 1a. The image was first converted to grayscale (Fig. 1b) and then preprocessed using image enhancement and background subtraction (see the Methods section) to obtain a binary image (Fig. 1c). Finally, the cells were segmented by the iCut algorithm (Fig. 1d). Although there were many touching cells, the iCut algorithm was able to segment all of them correctly even for a cluster of more than 5 touching cells. The corresponding NS1 image is shown in Fig. 2a with the enhancement shown in Fig. 2b. Next, the image was converted into a binary image (Fig. 2c) and the cells were segmented using the iCut algorithm (Fig. 2d). Despite heterogeneous brightness in the experimental image, most of the isolated cells and touching cells were correctly segmented.

### Comparison of segmentation results with those of different methods

We compared the iCut algorithm with two other state-of-the-art detection and segmentation algorithms, including the multi-scale LoG^8^ (MSL) and iterative voting^11^ (IV) algorithms. The parameters of the IV algorithm were *diameter* = 26, *min vote* = 500, and *sigma* = 3. The parameters of the MSL algorithm were the default parameters. For the gold standard, we manually marked each cell centroid and then calculated the recall, precision and F-score to evaluate (see the Methods section) the three algorithms. Recall is the ratio of the number of cells correctly detected by the algorithm to the number of manually marked cells. Precision is the ratio of the number of correctly detected cells to the total number of detected cells. F-score combines the precision and recall and is the harmonic mean of these two values.

**Figure 3:**
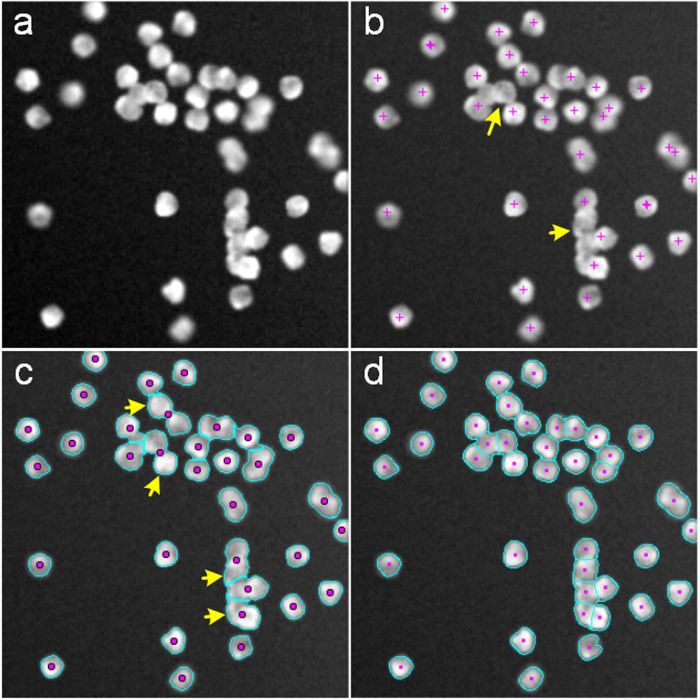
Comparison of different segmentation methods using a sub-image of the SIM1 image from the SIMCEP Benchmark_Set1 dataset. The pink points and crosses denote the cell centroids, and the cyan lines denote the partitioned cell contours. The yellow arrows denote incorrectly detected or segmented cells. (**a**) The sub-image of the SIM1 image. (**b**–**d**) The results obtained by the IV, MSL and iCut algorithms.

A comparison of the results for the SIM1 image is shown in Fig. 3 (only a sub-image is shown). The grayscale image of the sub-image is shown in Fig. 3a. The segmentation results of the IV, MSL and iCut algorithms are shown in Fig. 3b, c and d, respectively. The IV algorithm can only detect the cell centroid and cannot provide cell contour information. The IV and MSL algorithms fail to detect many cells and under-segment many cells, especially in regions containing touching cells. Strikingly, the iCut algorithm can segment most cells accurately. Quantitative comparisons for the SIMCEP Benchmark_Set1 dataset are listed in Table 1, including the recall, precision and F-score values for each image and the mean *μ* and standard deviation *σ* values across all the images. For the IV, MSL and iCut algorithms, the recall/precision values are 86.9 ± 2.7%/84.6 ± 1.9%, 73.2 ± 3.5%/87.3 ± 2.2% and 91.2 ± 2.1%/94.1 ± 1.8%, respectively. Thus, the precision, recall and F-score of the iCut algorithm are all higher than those of the two state-of-the-art algorithms.

**Figure 4:**
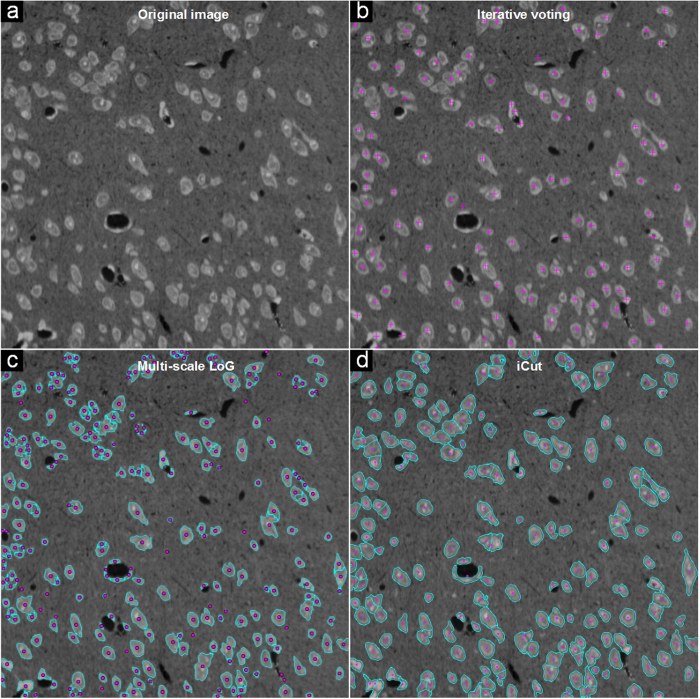
Comparisons of different segmentation methods for the NS1 images from the NSMOST dataset. The pink points and crosses denote the cell centroids, and the cyan lines denote the partitioned cell contours. (**a**) The original NS1 image. (**b**–**d**) The results obtained by the IV, MSL and iCut algorithms.

We also compared the iCut algorithm with the two algorithms using the NSMOST dataset. The results for the NS1 images are shown in Fig. 4. The IV algorithm fails to detect some cells, especially in regions where cells cluster. The MSL algorithm is sensitive to heterogeneous brightness in the image, and there is often under- or over-segmentation, particularly over-segmentation. The iCut algorithm can accurately segment most cells, although there are regions with many touching cells. Quantitative results are listed in Table 2. For the IV, MSL and iCut algorithms, the recall/precision values are 79.2 ± 7.4%/92.7 ± 3.6%, 94.5 ± 1.6%/56.7 ± 5.0% and 86.8 ± 4.1%/87.5 ± 5.7%, respectively. Here, the IV algorithm shows high precision but low recall, whereas the MSL algorithm shows high recall but low precision.

Student’s *t*-test was used to determine whether the F-score values of iCut differed significantly from those of the other two methods. Two *t*-test experiments were performed: (1) iCut algorithm vs IV algorithm and (2) iCut algorithm vs MSL algorithm. We first calculated the recall, precision and F-score values for the SIMCEP Benchmark_Set1 (Table 1) and NSMOST (Table 2) datasets. The *t*-test results for the F-score are presented in Table 3 at a confidence level of 0.05. The F-score of iCut is significantly higher than those of the two other algorithms for the SIMCEP Benchmark_Set1 dataset. For the NSMOST dataset, the F-score of iCut is significantly higher than that of MSL and not significantly different from that of IV (Table 3).

### Parameter analysis of the proposed algorithm

There is only one parameter in the iCut algorithm: the revised factor *S* (for details, see the Methods section). If *S* is too large, over-segmentation will occur; if *S* is too small, touching cells may not be segmented. The influence of changing the parameter in the proposed iCut algorithm was investigated. We considered the values *S* = {0.5, 1.0, 1.5, 2.0, 2.5, 3.0, 3.5, 4.0, 4.5} and ran the iCut algorithm. Then, we computed the recall, precision and F-score values at each parameter level for the two datasets. Only F-score was used to evaluate the parameter influence.

**Figure 5:**
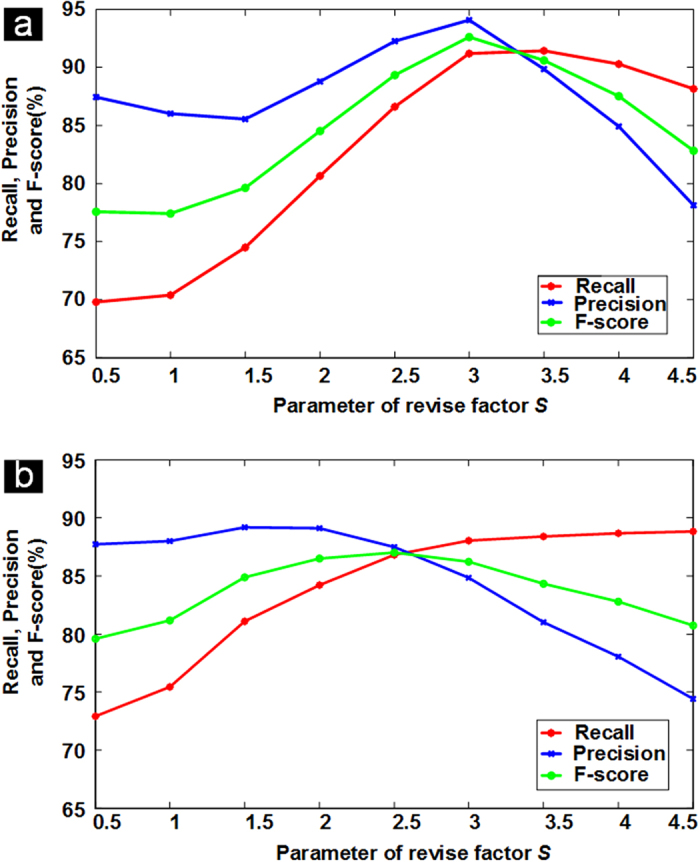
Parameter analysis performance of the iCut algorithm on the two datasets. The curves show the recall, precision and F-score values for different levels of the revised factor *S*. (**a**) The performance using the SIMCEP Benchmark_Set1 dataset. (**b**) The performance using the NSMOST dataset.

**Figure 6:**
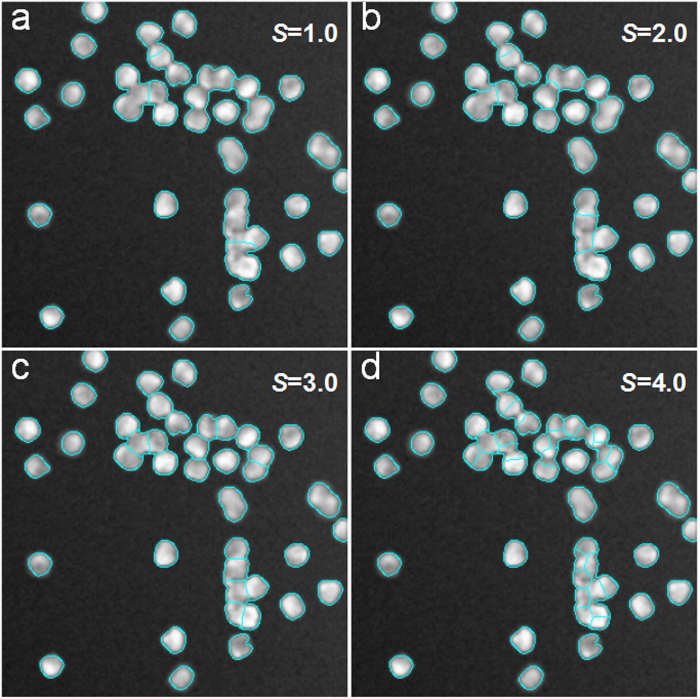
Parameter analysis of the iCut algorithm using the SIM1 image from the SIMCEP Benchmark_Set1 dataset. (**a**–**d**) The segmentation results with different parameter values: *S* = {1.0, 2.0, 3.0, 4.0}.

**Figure 7:**
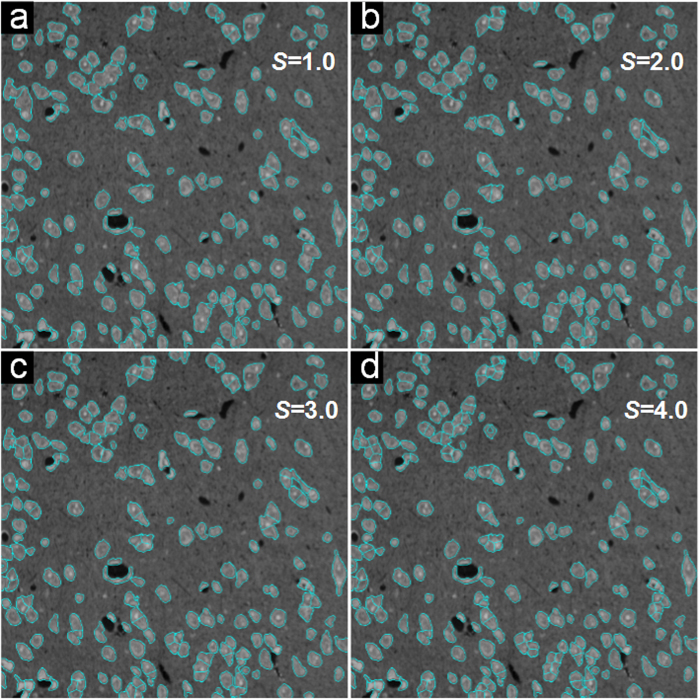
Parameter analysis of the iCut algorithm using the NS1 image from the NSMOST dataset. (**a**–**d**) The segmentation results with different parameter values: *S* = {1.0, 2.0, 3.0, 4.0}.

For the two datasets, we show the recall, precision and F-score values at different parameter levels in Fig. 5. The curves show that when the value of *S* is too large (*S* = 4.5) or too small (*S* = 0.5), the F-score performance is poor. When *S* = 2.5 − 3.5, the performance of F-score is optimal for the SIMCEP Benchmark_Set1 dataset, as shown in Fig. 5a. Similarly, when *S* = 2 − 3 the performance of F-score is optimal for the NSMOST dataset, as shown in Fig. 5b. In Fig. 6, we present the segmentation results with different values of *S* for the SIM1 image. When *S* = 1.0, there is substantial under-segmentation; many touching cells are not segmented in Fig. 6a. When *S* = 4.5, there is substantial over-segmentation; the number of segmented cells is greater than the number of real cells in Fig. 6c. A similar conclusion can be drawn from the segmentation results for the NSMOST dataset in Fig. 7. After a sanity check, parameter *S* can be fixed for batch analysis.

### Computational efficacy

The iCut algorithm was implemented in MATLAB R2010b (MathWorks, Natick, MA). We ran our code on a 64-bit Windows PC (CPU: Intel Core i5 at 3 GHz, RAM: 4 GB) and tested the execution time using the NSMOST dataset. The average time cost is approximately 23 s per image of 600 × 600 pixels. In comparison, the IV algorithm takes 8 s, and the MSL algorithm takes 20 s. The iCut algorithm has the highest computational time because it must solve an eigenvalue system with a time complexity *O*(*n*^*3*^), where *n* is the number of foreground pixels in the image. For tests with different SIMCEP Benchmark_Set1 images with different clustering probabilities, the time cost tends to increase as the clustering increases. With the power of the Parallel Computing Toolbox of MATLAB, a parallel version of the iCut program can increase the speed up to 2-fold using the same PC hardware configuration. Both the single thread and parallel version programs of iCut are freely available upon request.

## Discussion

Here, we have proposed iCut, an intervening and concave contour-based normalized cut algorithm, for the segmentation of touching cells in 2D images. This is a companion paper to our previous work on partitioning touching cells in 3D space^7^. The iCut algorithm creatively encodes spatial location and intervening and concave contour information into a weighting matrix, which enables the normalized cut algorithm to accurately separate touching cells into isolated cells. Although there are many touching cells in the SIMCEP Benchmark_Set1 and NSMOST datasets, iCut is comparatively effective and achieved a recall/precision of 91.2 ± 2.1%/94.1 ± 1.8% and 86.8 ± 4.1%/87.5 ± 5.7% for these datasets, respectively. To balance the recall and precision, F-score, a harmonic mean of the recall and precision, was used to evaluate the performance of our algorithm. Statistical analysis showed that the F-score of the iCut algorithm is usually higher than those of the two state-of-the-art algorithms for the two tested datasets (Table 3). Of course, iCut also works well for isolated cells, making iCut a flexible algorithm for cell detection and segmentation.

**Figure 8:**
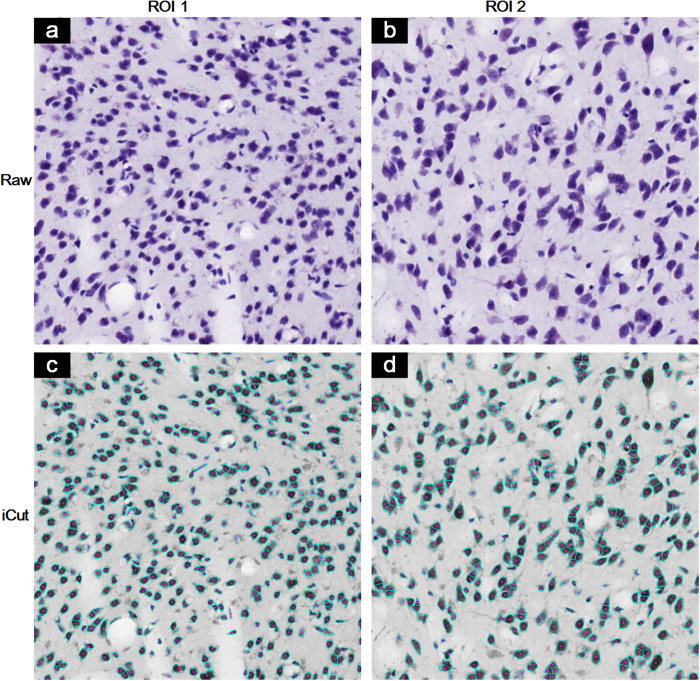
Pilot test of the iCut algorithm using conventional Nissl-staining brain slice dataset from brainmaps.org. Two arbitrary regions of interest (ROI) from a coronal brain section of 20 μm thick are shown as raw images in (**a**) and (**b**). iCut achieves F-score as 89.7% and 87.5% in the segmentation results of (**c**) and (**d**) respectively.

Heterogeneous brightness in optical images and the irregular geometric characteristics of touching cells have posed challenges for many algorithms^8^,^11^. The IV algorithm is based on the projection along the radial direction and is sensitive to parameter selection. The MSL algorithm produces substantial over-segmentation due to heterogeneous brightness in the NSMOST dataset. Similar to the over-segmentation that occurs when a cell has chromatin texture^8^, this over-segmentation is presumably related to the LoG filtering, which could produce a local maximum for determining centroids. The iCut algorithm runs on a binary image and thus, to a certain extent, avoids the influence of heterogeneous brightness. Cells often possess irregular geometric and clustering characteristics, and these are likely to result in segmentation errors. Visual inspection of the NSMOST dataset indicates that some cells located in the vessel wall resemble crescents^24^. Additionally, because of the thinness of the slice (2 μm thick), some cells are only imaged with a small cross section, i.e., these cells are apparently small. Because the construction of the weighting matrix of iCut is not sensitive to cell shape and size, iCut facilitates the robust segmentation of touching cells. The proposed iCut algorithm differs from traditional cell segmentation algorithms that work directly at the intensity and gradient levels. iCut works at the cell-group level, which is more correlated with the whole cell itself and is thus less sensitive to brightness variations inside the cells. Including the MSL algorithm^8^, some other methods also exploit background normalization and thresholding. The better performance of iCut not only depends on the preprocessing procedure but also benefits from the design of the weighting matrix for the normalized cut algorithm. In addition to being used for the two datasets tested in this paper, the iCut algorithm can be applied to other datasets that have cellular resolution. In fact, iCut has been tested using a conventional Nissl-stained slice (20 μm thick) and obtained high accuracy in Fig. 8. iCut achieves F-score over 87.5% in the real tissue sample. In principle, iCut can segment other blob-like objects in a digital image.

To counteract the time complexity of the iCut algorithm, we will use C++ programming to parallelize iCut in the future to improve its time efficacy compared with that for the parallel implementation in MATLAB. The era of big data of neuroimaging is upon us^20^. Our automated, precise method should be very helpful for elucidating brain cytoarchitecture characteristics, such as cell number, size, location, density and spatial distribution^4^,^24^,^25^. We believe that iCut will promote brain research using quantitative histology.

## Methods

### Datasets

**Figure 9:**
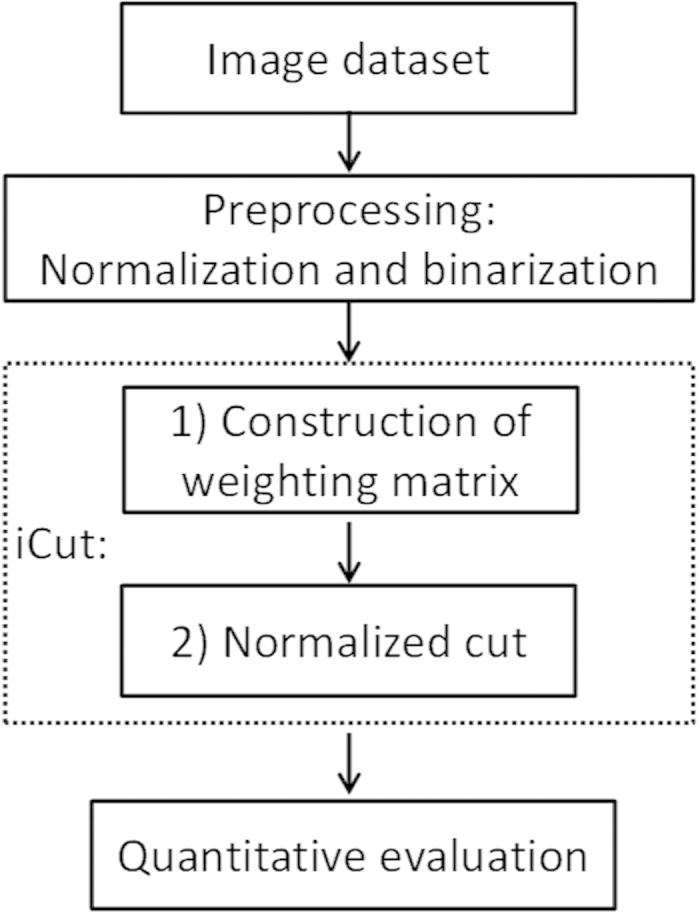
An outlined workflow of this study. Image dataset is preprocessed as input for iCut. iCut involves two key steps: (1) construction of weighting matrix and (2) Implementaiton of normalized cut. And quantitative evaluation is done for assess the performance of iCut.

We performed the experiments using three datasets: the publicly available SIMCEP benchmark^21^, the NSMOST dataset obtained using a Nissl-staining and micro-optical sectioning tomography imaging system^22^, and the conventional Nissl-staining brain slice dataset from brainmaps.org^23^. An outlined workflow of this study with image dataset as input was shown in Fig. 9.

The SIMCEP benchmark dataset has three sets of synthetic images and is designed for the validation of cell image analysis algorithms. We used Set 1 (“Clustering with increasing probability”) of this dataset and named it SIMCEP Benchmark_Set1. Set 1 contains 20 simulated fluorescence microscopy images at five levels of cell clustering (touching). We chose images with the following simulation parameters: probability of clustering *p* = 0.3 and overlap *L* = 1. All 20 images, named SIM1 to SIM20, were converted to grayscale images, and each image was 950 × 950 pixels and contained 300 cells.

The NSMOST dataset comprises the micro-optical imaging of a C57BL/6 mouse brain. The mouse brain was Nissl stained^24^, and the dataset was obtained using a micro-optical sectioning tomography system with a pixel resolution of 0.35 × 0.4 μm. This dataset is complicated due to the background noise, varied cell morphology and heterogeneous brightness. The brightness between adjoining slices was corrected^26^. There are a total of 20 images, termed NS1 to NS20, and each image is 600 × 600 pixels.

The real slice dataset from brainmaps.org is one whole coronal mouse brain section 20 μm thick with a pixel resolution of 0.46 × 0.46 μm. Only two 600 × 600 pixels ROIs were extracted for the pilot testing of iCut.

### Image preprocessing

Before segmentation, the image is preprocessed using image enhancement, background subtraction, binarization, and noise elimination. Inconsistent illumination in some regions of an imaging system may cause heterogeneous brightness; thus, it is necessary to enhance the image contrast. Here, the image is enhanced in a linear stretch manner: suppose that *r*_*min*_ and *r*_*max*_ denote the minimum and maximum intensity levels, respectively, in the original image. A transformation function (using the *imadjust* function in MATLAB) stretches the levels linearly from the original range (*r*_*min*_, *r*_*max*_) to the full range.

After image enhancement, the background of the cell is subtracted from the image. To separate the cell target from the background, we perform image binarization using the Otsu algorithm^27^. Afterward, some background noise may be regarded as the foreground; thus, noise that has a small area is eliminated. To calculate the area, a connected component in the binary image is extracted by labeling adjacent pixels (using the *bwlabeln* and *regionprops* functions in MATLAB), and the area of a connected component is the number of these adjacent pixels.

### Intervening and concave contour-based normalized cut algorithm

For an isolated cell, each connected component in the preprocessed image represents only one cell; for a cluster of touching cells, each connected component represents several cells. The latter must be segmented into individual isolated cells. Herein, we develop an intervening and concave contour-based normalized cut algorithm derived from the original normalized cut algorithm^28^. The proposed algorithm considers image segmentation as a graph partition problem (cut, partition and segment have the same meaning in this paper). iCut constructs a graph *G* = (*V*, *E*, *W*) with the image pixel as graph node *V*, the connection between pixels as graph edge *E*, and the similarity between pixels as weight *W*. The proposed algorithm includes two steps: (1) constructing the weighting matrix *W* of the graph and (2) cutting the graph into different groups with each group corresponding to an isolated cell.

#### (1) Constructing the weight of graph

The weight *W* reflects the likelihood of the pixels being partitioned into one group; the brightness, spatial location, color and texture information are often used to construct *W*. We use three types of information: spatial location, intervening contours and concave contours, which are defined as follows.

Spatially adjacent pixels are likely to belong to one group; thus, the spatial location weight *W*_*s*_ is defined as follows:


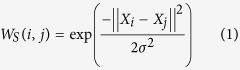


where *X*_*i*_ and *X*_*j*_ denote the pixel spatial location, and this information regarding connecting adjacent pixels is very useful. When two cells are touching, we may partition them into one group using only the spatial location information. Thus, intervening and concave contour cues are necessary for better segmentation.

Intervening contours are identified as described in the literature^29^ and correspond to edges in the image. A concave contour occurs at the junction points of two touching cells^15^. The main step in identifying a concave contour is calculating the concaveness values of all the contour points; then, a threshold is applied to these concaveness values. The concaveness value of a contour point, *p* (*m*, *n*), is calculated as follows:


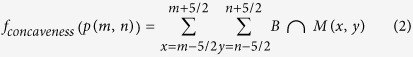


where *B* is the binary image and *M* is a 5 × 5 mask centered on *p* (*m, n*). The concaveness value of the contour point*, f*_*concaveness*_(*p*(*m, n*)), is the number of pixels of *M* that intersects *B.* To obtain a concave contour, *C*, we threshold the concaveness values of all contour points, *f*_*concaveness*_(*p*), by *T* (*T* = 0.65, a slightly higher threshold also works):


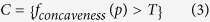


Next, the intervening and concave contour information is encoded into the weight *W* in a manner similar to that used by Cour *et al.*^29^ We define an affinity weight *W*_*c*_ by measuring the intervening and concave contour between two pixels:


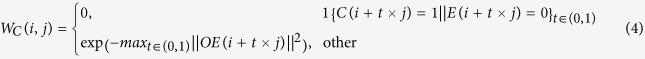


where *i*, *j* denote the pixel location, (*i* + *t* × *j*) denotes a straight line joining pixels *i* and *j*, and *C*(*i* + *t* × *j*) = 1 is used to judge whether the straight line intersects a concave contour. *E* is an edge in the image.* E*(*i* + *t* × *j*) = 0 is used to determine whether the straight line intersects an image edge, and this intersecting edge is the intervening contour. 1{●} means that if the expression in the brackets is true, the result is true. Specifically, if the straight line intersects an intervening contour or a concave contour, *W*_*c*_(*i*, *j*) = 0. *OE* in Eqn (4) is called the oriented energy, and it is obtained through the convolution of an image and oriented filter pair to extract the magnitude of the edge responses^30^. The term “*max*_*t*∈(0,1)_||*OE*(*i* + *t* × *j*)||^2^” denotes the maximum value of an oriented energy that intersects the straight line, and *W*_*c*_(*i*, *j*) is low if *i*, *j* are on two sides of an edge, i.e., one is a foreground pixel and the other is a background pixel in the binary image.

The oriented energy *OE* at angle 0° is defined as follows^31^:


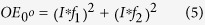


where


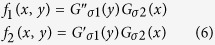


where *I* is the image before the convolution and *G*_*σ*_(*x*) is a Gaussian with a standard deviation *σ*. The maximum value of *OE* at angle 0° is on the horizontal contour. The two filter kernels *f*_1_ and *f*_2_ in Eqn (5) can be rotated to various angles, and non-maximal suppression can be used to obtain the dominant orientation energy. The definition of *OE* above is based on the grayscale image. In the binary image, *B*, the *OE* of the foreground and background is consistent, except the contour. Thus, we do not need to filter using multiple angles, and we can use a simple Gaussian to transform Eqn (5) into the following:


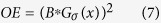


and Eqn (4) is transformed into the following:


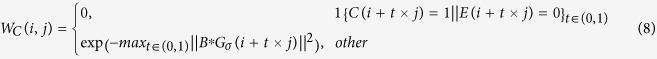


The total weight *W* of the graph *G* is as follows:


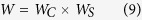


**Figure 10:**
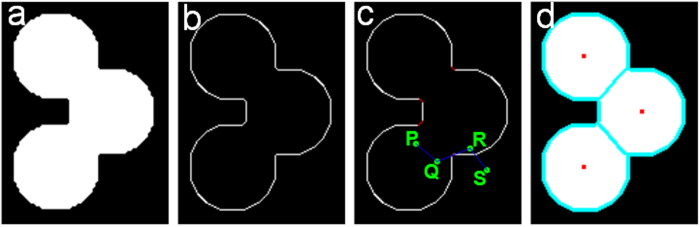
The intervening and concave contours of a synthesized cell image and the iCut segmentation result. (**a**) The synthesized binary image with three touching cells. (**b**) An edge in the binary image. (**c**) The intervening and concave contours. The red points are the concave contours. The straight line joining pixels *P* and *Q* does not intersect the contour; thus, *W*_*C*_(*P*, *Q*) ≠ 0. The straight line joining pixels *R* and *Q* intersects the concave contour; thus, *W*_*C*_(*R*, *Q*) = 0. The straight line joining pixels *R* and *S* intersects the intervening contour; thus, *W*_*C*_(*R*, *S*) = 0. (**d**) The iCut result along with the cyan line showing the contour of the partitioned groups. The red points are the centroids of each group and are also the cell centroids.

We use a synthesized binary image with three touching cells to explain the intervening and concave contour more clearly (Fig. 10a). The edge image is shown in Fig. 10b. The intervening and concave contours are shown in Fig. 10c, where the red points are the concave contour obtained using Eqns (2,^3^). We show four pixels, *P*, *Q*, *R*, and *S*, in the image. The straight line joining *R* and *S* intersects an edge (intervening contour), and the straight line joining *R* and *Q* intersects a concave contour; thus, *W*_*C*_(*R*, *Q*) = *W*_*C*_(*R*, *S*) = 0. Then, in the subsequent normalized cut step, pixels *P* and *Q* will be partitioned into one group, pixels *R* and *Q* will be partitioned into different groups, and pixels *R* and *S* will be partitioned into different groups.

#### (2) Normalized cut

After constructing the weight of graph *G*, we must cut the graph into different groups. Suppose *G* is cut into two groups *A* and *B*, subject to *A* ∪ *B* = *V* and *A* ∩ *B* = *φ*. The connection between groups *A* and *B* describes the similarity of the two groups, and the cut between them is as follows:


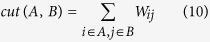


where *W*_*ij*_ is the weight linking nodes *i* and *j*. The partitioning or cut of the graph corresponds to a minimization of the sum of all the weights between different groups, i.e., minimum *cut*(*A*, *B*), and an optimization algorithm^29^ has been proposed to solve the problem. However, this optimization algorithm is likely to generate small groups, in some cases just a node (this situation is called “*unnatural bias*”). To avoid this situation, Shi and Malik^28^ proposed a new algorithm to measure the dissimilarity between different groups, i.e., they used the degree of dissimilarity of groups *A* and *B* as a penalty when weighting the graph. This new algorithm is called normalized cut (Ncut):


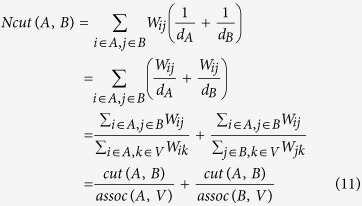


where *assoc*(*A*, *V*) is the total connection from *A* to *V* of the graph. Given this penalty term, if the small group *A* has few nodes, then *assoc*(*A*, *V*) will have a small value, and the value of *Ncut*(*A*, *B*) will be very large; this result follows the principle of minimum *Ncut*(*A*, *B*). Thus, Ncut can avoid the “*unnatural bias*” problem.

We discuss how to optimize the normalized cut. Suppose *x* is an *N* = |*V*| dimensional indicator vector. The indicator value is *x*_*i*_ = 1 if node *i* is in group A; otherwise, *x*_*i*_ = −1. Define *d*(*i*) *=* *∑*_*j*_*W*_*ij*_ as the sum of the weights between nodes *i* and *j*. *D* is a diagonal matrix with *d*(*i*) on the diagonal. The optimal cut can be found by solving the following minimum problem:


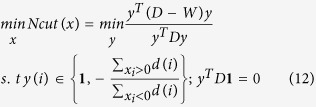


Relaxing *y* in Eqn (10) to take on real values, we obtain a generalized eigenvalue system:


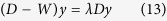


The detailed solution of the system is described in the literature^28^. After the eigenvectors of Eqn (11) are computed, the second smallest eigenvector is used to partition (cut) the graph *G* into two groups *A* and *B*. Next, the cut is recursively run on the partitioned groups until reaching a stop condition. We chose the maximum recursion number *k* as the stop condition. The value of *k* is determined to be *k* *=* *S* × (*N* + 1), where *N* denotes the number of concave points and *S* is a revised factor. The revised factor is introduced because the normalized cut may occur in the background; thus, the algorithm wastes one recursion and must be revised to include additional recursions.

The time complexity of this algorithm is very great, and the Lanczos method is often used to reduce the complexity. For the binary image, we exploit another strategy: each time, only one connected component is processed. The detailed steps of the iCut algorithm are described below:

- Detect concave points and obtain a concave contour;
- Construct the weighting matrix *W* using the spatial location and the intervening and concave contour information;
- Solve the generalized eigenvalue system in Eqn (13).
- Use the eigenvectors of the system to cut or partition the graph.
- Recursively run steps (*c*-*d*) on the partitioned graph until the number of recursions equals *k*.

We ran the iCut algorithm on the synthesized image with touching cells, and the result is shown in Fig. 10. The cyan contour in Fig. 10d is the partition result, and the touching cells have been partitioned into many groups with each group corresponding to a cell. After the graph is partitioned, the centroid of the group is the cell centroid, which is shown in Fig. 10d.

### Quantitative evaluation

The segmentation result is evaluated quantitatively as previous work^14^,^32^. The performance of the results can be described in term of accuracy. Researchers assess accuracy using two methods: pixel-based and nucleus-based evaluations. The pixel-based method assesses the accuracy in terms of the pixel areas of a correctly segmented result, and it depends mainly on the binarization method instead of the iCut algorithm; thus, we do not use the pixel-based method. The nucleus-based method assesses the accuracy in terms of the number of correctly segmented cells and is suitable for the iCut algorithm to assess the accuracy of segmenting touching cells; thus, we employ the nucleus-based method.

In nucleus-based evaluations, a segmented cell is matched to a manually marked cell if the distance between their cell centroids is less than the cell radius *R* (*R* = 6 μm in this paper). Then, the segmented and manually marked cells form one unique pair (*one-to-one matches*). Assuming that the number of the manually marked cells is *N*_*g*_, the number of the automatic segmented cells is *N*_*d*_, and *N*_*m*_ automatically segmented cells can be a *one-to-one match* with a manually marked cell, then the recall, precision, and F-score are calculated as follows^32^:


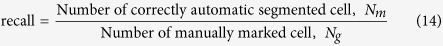



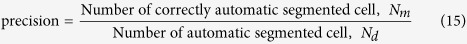



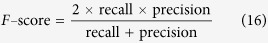


## Additional Information

**How to cite this article**: He, Y. *et al.* iCut: an Integrative Cut Algorithm Enables Accurate Segmentation of Touching Cells. *Sci. Rep.* **5**, 12089; doi: 10.1038/srep12089 (2015).

## Figures and Tables

**Table 1:** Performance of iCut and other algorithms using the SIMCEP Benchmark_Set1 dataset.

Image slice	IV			MSL			iCut		
	Recall	Precision	F-score	Recall	Precision	F-score	Recall	Precision	F-score
1	88.4	83.5	85.9	74.4	87.9	80.6	92.5	95.1	93.8
2	82.9	84.9	83.9	68.5	85.8	76.2	89.7	92.6	91.1
3	83.7	84.9	84.3	69.4	84.7	76.3	92.0	96.4	94.1
4	84.3	81.5	82.9	71.0	86.3	77.9	90.8	94.0	92.4
5	89.8	86.0	87.9	70.3	84.4	76.7	94.2	95.2	94.7
6	90.0	84.7	87.3	78.0	89.5	83.4	90.9	94.4	92.6
7	91.9	86.1	88.9	78.1	90.6	83.9	92.3	95.1	93.7
8	87.0	85.5	86.2	71.2	86.2	78.0	91.3	94.8	93.0
9	90.2	88.0	89.1	76.6	90.8	83.1	94.6	98.2	96.4
10	88.5	84.1	86.2	75.7	88.6	81.6	88.9	89.5	89.2
11	86.6	86.6	86.6	72.8	87.3	79.4	91.2	92.1	91.6
12	89.1	85.9	87.5	81.0	92.2	86.2	94.6	95.5	95.0
13	88.8	85.8	87.3	72.7	87.0	79.2	87.4	93.6	90.4
14	87.0	83.6	85.3	74.6	88.1	80.8	91.3	93.5	92.4
15	84.4	86.7	85.5	73.8	88.2	80.4	89.1	94.6	91.8
16	87.8	83.5	85.6	73.6	86.9	79.7	89.2	92.8	91.0
17	87.2	82.3	84.7	73.3	87.2	79.6	93.4	94.1	93.7
18	83.5	84.6	84.0	67.6	84.2	75.0	88.4	93.3	90.8
19	84.1	84.4	84.2	70.0	85.7	77.1	89.3	92.8	91.0
20	83.4	79.6	81.5	72.1	85.3	78.1	92.4	93.4	92.9
*μ* ± *σ*	86.9 ± 2.7	84.6 ± 1.9	85.7 ± 1.6	73.2 ± 3.5	87.3 ± 2.2	79.7 ± 2.4	91.2 ± 2.1	94.1 ± 1.8	92.6 ± 1.5

**Table 2:** Performance of iCut and other algorithms using the NSMOST dataset.

Image slice	IV			MSL			iCut		
	Recall	Precision	F-score	Recall	Precision	F-score	Recall	Precision	F-score
1	70.2	96.2	81.2	94.9	60.0	73.5	91.6	90.1	90.8
2	79.1	94.9	86.3	95.7	50.3	65.9	85.0	92.4	88.5
3	71.5	91.6	80.3	92.7	59.5	72.5	90.2	89.2	89.7
4	79.9	89.3	84.3	96.4	55.9	70.8	79.1	84.0	81.5
5	83.6	90.5	86.9	96.1	59.2	73.3	87.5	93.3	90.3
6	85.4	90.9	88.1	95.7	58.1	72.3	88.4	92.4	90.4
7	86.7	87.0	86.8	95.6	54.0	69.0	86.7	85.4	86.0
8	83.2	95.8	89.1	94.6	54.7	69.3	81.2	84.6	82.9
9	80.6	94.4	87.0	94.1	66.5	77.9	85.3	93.5	89.2
10	78.0	87.1	82.3	91.8	50.1	64.8	81.1	81.1	81.1
11	84.0	93.0	88.3	95.3	57.5	71.7	82.2	88.0	85.0
12	63.1	97.9	76.7	94.9	49.8	65.3	82.2	88.9	85.4
13	60.8	100	75.6	94.0	54.5	69.0	86.4	92.5	89.3
14	82.1	92.2	86.9	95.7	69.6	80.6	84.8	92.9	88.7
15	79.9	95.5	87.0	94.7	57.1	71.2	90.5	87.7	89.1
16	83.0	92.4	87.4	93.6	59.3	72.6	92.6	84.5	88.4
17	77.3	90.7	83.5	89.7	57.1	69.8	88.6	77.4	82.6
18	86.5	93.7	90.0	95.7	56.0	70.7	92.0	92.6	92.3
19	86.3	86.4	86.3	93.5	52.5	67.2	90.2	72.3	80.3
20	83.9	94.7	89.0	95.7	52.8	68.1	90.3	87.0	88.6
*μ* ± *σ*	79.2 ± 7.4	92.7 ± 3.6	85.1 ± 3.4	94.5 ± 1.6	56.7 ± 5.0	70.8 ± 3.0	86.8 ± 4.1	87.5 ± 5.7	87.0 ± 3.3

**Table 3:** *t*-test results for iCut vs IV and iCut vs MSL comparisons of F-score at confidence level = 0.05.

Dataset	*t*-test result	vs IV	vs MSL
SIMCEP Benchmark_Set1	Significance	+	+
	*p*-value	5.24E-14	2.78E-19
NSMOST	Significance	−	+
	*p*-value	0.13	3.38E-16

“+” indicates a significant difference, and “−” indicates no significant difference.
